# Exploring Barriers and Protective Factors in Decision Fatigue Among Clinical Nursing Managers During the COVID‐19 Pandemic: A Qualitative Content Analysis

**DOI:** 10.1155/jonm/5553282

**Published:** 2026-06-28

**Authors:** Sarah Sheikh, Fatemeh Hajibabaee, Mohammad Ali Cheraghi, Alireza Nikbakht Nasrabadi, Maryam Esmaeili

**Affiliations:** ^1^ Department of ICU Nursing, School of Nursing and Midwifery, Nursing and Midwifery Care Research Center, Tehran University of Medical Sciences, Tehran, Iran, tums.ac.ir; ^2^ Nursing and Midwifery Care Research Center, School of Nursing and Midwifery, Tehran University of Medical Sciences, Tehran, Iran, tums.ac.ir; ^3^ School of Nursing and Midwifery, Tehran University of Medical Sciences, Tehran, Iran, tums.ac.ir; ^4^ Tehran University of Medical Sciences and Al-Subtain Univetsity of Medical Sciences, International Branch of Tehran University of Medical Sciences, Karbala, Iraq

**Keywords:** conventional content analysis, COVID-19 pandemic, crisis management, decision fatigue, nursing managers

## Abstract

**Background and Objective:**

Decision fatigue of nursing managers in stressful hospital environments is considered one of the fundamental challenges of healthcare management. It affects the quality of decisions and organizational outcomes. This study aimed to explore both the barriers that exacerbate decision fatigue and the protective factors and strategies that mitigate it from the perspective of clinical nursing managers during the COVID‐19 pandemic.

**Methodology:**

This qualitative study was conducted with a conventional content analysis approach. Participants consisted of 11 nursing managers working in the hospitals affiliated with Tehran University of Medical Sciences in Iran, who had been selected purposively and theoretically. Data were collected through semistructured, individual, and face‐to‐face interviews. MAXQDA software Version 18 was used for data management. Data validity was ensured according to the criteria of Lincoln and Guba (1985).

**Results:**

The results revealed three main categories that encompassed both barriers (e.g., responsibility overload, organizational pressures) and protective factors (e.g., humanistic leadership, utilization of support resources), including organizational decision‐making and leadership strategies, communication management and organizational interactions, and individual and organizational patterns in decision‐making. Each of these categories consisted of two subcategories, including decision‐making regulation and guidance, and humanistic management and leadership for the first category, activation of interaction and communication processes, and utilization of support resources for the second category, and finally independence and responsibility in decision‐making, and organizational interest and commitment for the third category.

**Conclusion:**

Decision fatigue of nursing managers during the COVID‐19 pandemic was shaped by a dynamic interplay of barriers and protective strategies. Clarifying these dual influences provides a coherent framework for understanding managerial decision‐making under crisis conditions. Strengthening delegation, implementing decision pilots, and building organizational trust are effective strategies for reducing cognitive pressure and improving the quality of decision‐making. Medical centers are suggested to reduce decision fatigue by addressing barriers such as organizational pressures and responsibility overload, while simultaneously strengthening protective factors such as supportive leadership, effective communication, and organizational trust.

## 1. Introduction

Since the beginning of the 21st century, emerging diseases from the coronavirus family have suddenly appeared, with COVID‐19 being the most widespread and disruptive [1]. This disease, with high mortality and morbidity, became a global pandemic in less than 4 months [2, 3]. The widespread consequences of this pandemic affected almost all vital aspects of societies and caused widespread changes in the health, economic, political, social, cultural, and educational domains [4, 5]. Meanwhile, hospital systems, as one of the most important pillars of healthcare, faced numerous challenges, including shortages of human resources and protective equipment, implementation of protective policies for employees, cancellation of staff leaves, increased demand for intensive care beds and mechanical ventilation, and rapid expansion of hospital capacities [6, 7]. These critical conditions significantly increased the responsibilities of nursing managers and forced them to make critical decisions in the shortest possible time while managing workforce shortages, reducing staff anxiety, implementing new guidelines, reorganizing wards, and ensuring safe care environments [8, 9]. In such circumstances, managerial decisions played a key role in maintaining hospital performance, but the ongoing challenges of the pandemic placed substantial cognitive and emotional pressure on managers, affecting the accuracy and timeliness of their decisions. This led to decision fatigue in managers and reduced their efficiency. Decision‐making is considered one of the most important tasks of nursing managers and is the essence of management [10, 11]. Nursing managers must be able to make quick, efficient, and logical decisions in critical situations, because any delay or mistake in decision‐making can have irreparable consequences [12]. However, studies have shown that high workload, job stress, and the need for making multiple decisions in critical situations contribute to decision fatigue, which in turn impairs managerial performance and reduces clinical efficiency [13, 14]. Decision fatigue leads to passive and impulsive behaviors and impairs the quality of decision‐making [15, 16]. This concept is derived from the self‐control power model proposed by Baumeister et al. [17]. This model suggests that humans have a limited capacity to regulate behavior and process information and, when their mental resources are depleted, they experience decreased executive function, poor emotional regulation, delayed decision‐making, and impulsive decisions [13, 18, 19]. Studies have shown that decision fatigue among frontline nurses increases significantly during health crises, including COVID‐19, and this negatively affects their quality of care and mental health [20]. A study conducted by Pignatelli and colleagues also showed that decision fatigue can lead to reduced accuracy in clinical decision‐making and increased job stress [15].

Despite extensive evidence that exists regarding decision fatigue and its effects on nursing managers’ performance, few studies have examined the actual experiences of nursing managers in facing decision‐making challenges during the COVID‐19 crisis [21]. A study by Vázquez‐Calatáyod et al. found that nursing managers had to make frequent, abrupt, and nonconsensual decisions during the COVID‐19 pandemic, and this increased their uncertainty and decreased their self‐confidence. This study also noted that some managers believed they were unable to make correct and effective decisions due to a lack of access to up‐to‐date information about new guidelines [22]. On the other hand, some studies have shown that decision fatigue gradually increases over time and has a negative impact on the quality of management decisions in the long term [23]. In a study by Allan et al., the timely need for consecutive decision‐making during the COVID‐19 pandemic led individuals to make conservative, costly, and inefficient decisions [24]. In addition, a study on time management and self‐leadership in nursing managers argued that nursing managers should adopt effective strategies to reduce decision‐making pressure and manage stress in critical environments [25]. Given that more than four years have passed since the outbreak of the COVID‐19 pandemic, identifying effective strategies to reduce decision fatigue in nursing managers seems essential to mitigate ongoing challenges and strengthen managerial preparedness for future crises. Also, since limited studies have addressed this aspect of management in nursing, and also considering that qualitative studies only focus on the real experiences of participants and provide valuable insights into the challenges, the present study was conducted with a qualitative approach to explore the factors influencing decision fatigue—including both barriers and protective factors—from the perspective of clinical nursing managers during the COVID‐19 pandemic.

## 2. Methodology

A conventional content analysis approach was employed to explore the factors influencing decision fatigue—including both barriers and protective factors—among clinical nursing managers during the COVID‐19 pandemic. This study was reported in accordance with the Consolidated Criteria for Reporting Qualitative Research (COREQ) checklist. This qualitative method facilitated an in‐depth exploration of participants’ experiences, enabling the identification of latent themes and contextualized insights within managerial decision‐making processes.

### 2.1. Study Design

This qualitative study was conducted using a conventional content analysis approach. This approach allowed for the inductive extraction of patterns, themes, and categories from narrative data, supporting a contextually grounded interpretation of the phenomenon.

### 2.2. Research Setting

The study was conducted in teaching hospitals affiliated with Tehran University of Medical Sciences. These publicly funded healthcare institutions offer a broad spectrum of specialized and subspecialized medical services and serve as primary centers for clinical education and training. The selected hospitals provided a diverse and dynamic environment, reflecting the organizational and managerial contexts relevant to the study objectives.

### 2.3. Participants and Sampling

This study employed purposive and theoretical sampling strategies over a 14‐month period (2022–2024) to recruit nursing managers across varying hierarchical levels (senior, intermediate, and novice) with diverse demographic profiles. Participants consisted of 11 nursing managers working in hospitals affiliated with Tehran University of Medical Sciences in Iran. The primary sampling strategy was purposive sampling, which ensured inclusion of managers with diverse demographic and professional backgrounds relevant to the study aim. Theoretical sampling was later used selectively to refine emerging categories during analysis.

In addition, the snowball technique was used to identify further participants through recommendations from initial interviewees. This combination enhanced both the breadth and depth of managerial perspectives. A total of 11 participants were included: six head nurses, three nursing supervisors, one hospital matron, and one university‐level nursing administrator.

To ensure heterogeneity in perspectives, sampling considerations included age, clinical and managerial experience, marital status, number of children, and educational attainment. The initial participant was selected based on a demonstrated ability to make effective decisions in high‐pressure scenarios, as confirmed by peer and senior nursing manager recommendations. Sampling proceeded iteratively until data saturation was achieved.

Inclusion criteria specified a minimum of six months’ experience in a clinical nursing management role (head nurse, supervisor, or matron) during the COVID‐19 pandemic, along with a willingness to participate in the study.

### 2.4. Data Collection

Data were collected through semistructured, in‐depth interviews designed to elicit rich, narrative accounts of participants’ experiences. Interviews commenced with open‐ended questions, including the following:

• “Can you describe an example of your management decisions during the COVID‐19 pandemic?”
• “In what situations did you feel that the quality of your decision‐making was impaired?”
• “What factors prevented or facilitated you in managing decision fatigue?”
• “What obstacles did you experience in the decision fatigue process?”

Follow‐up probing questions—such as “Can you explain more?” and “What do you mean by that?”—were used to clarify and deepen responses based on participant feedback. At the conclusion of each session, participants were invited to share any additional relevant experiences not previously discussed.

Each interview lasted approximately 60–90 min and was audio‐recorded with informed consent. Supplementary field notes were taken to capture contextual observations and nonverbal cues. Interviews were conducted in participants’ offices during working hours, based on individual preferences and logistical feasibility. Data collection continued until thematic saturation was reached after eight interviews, with three additional interviews conducted to confirm saturation and enhance analytical robustness.

### 2.5. Data Analysis

Data analysis was conducted using the conventional content analysis approach described by Graneheim and Lundman [26]. Immediately after each interview, the audio‐recordings were transcribed verbatim and read several times to achieve immersion in the data. Meaning units relevant to the study aim were identified and subsequently condensed while preserving their core content. These condensed meaning units were then coded inductively. The initial codes were compared for similarities and differences and were grouped into subcategories. Through further abstraction, subcategories were organized into broader categories that captured the underlying patterns in the data. The analytic process was iterative, with continuous review and refinement to ensure internal consistency and alignment with participants’ experiences. No “core category” was generated, as this step is not part of conventional content analysis.

MAXQDA Version 18 software was utilized to organize, manage, and support the coding process and data analysis, ensuring methodological rigor and transparency in handling the large volume of qualitative data.

### 2.6. Trustworthiness of the Data

To ensure the rigor of the qualitative findings, Lincoln and Guba’s [27] criteria for trustworthiness—credibility, dependability, confirmability, and transferability—were employed.

• *Credibility* was enhanced through the prolonged engagement of the primary researcher in iterative data analysis and comparative review. Additionally, the involvement of two independent qualitative research experts external to the study team contributed to the validation of emerging interpretations.
• *Dependability* was supported by member checking and peer debriefing, wherein participants and research colleagues reviewed the coding processes and thematic conclusions to ensure analytical consistency and interpretive accuracy.
• *Confirmability* was established through an external audit process. The finalized findings were presented to four faculty members with expertise in qualitative inquiry, who independently confirmed the validity of the interpretations. Maximum variation sampling was also applied to capture a broad range of perspectives and enhance the generalizability of insights.
• *Transferability* was strengthened by maintaining a comprehensive audit trail throughout the research process. Detailed records of methodological decisions, coding frameworks, and analytical memos were documented to enable external review and replication by future researchers.

### 2.7. Findings

The participants’ ages ranged from 35 to 60 years, with a mean age of 45 years. Detailed demographic characteristics of the study participants are presented in Table 1.

**TABLE 1:** Demographic characteristics of the participants.

Code	Position	Age	Work experience	Management experience	Marital status	Number of children	Education
P1	Head Nurse	40–45	18	6	Married	0	Master’s degree
P2	Head Nurse	35–40	15	6	Married	1	Master’s degree
P3	University Nursing Director	55–60	33	28	Married	2	Master’s degree
P4	Head Nurse	45–50	33	8	Married	2	Bachelor’s degree
P5	Supervisor	40–45	15	5	Married	2	Bachelor’s degree
P6	Head Nurse	35–40	12	4	Married	1	Bachelor’s degree
P7	Head Nurse	40–45	18	6	Married	2	Master’s degree
P8	Supervisor	45–50	24	10	Married	2	Doctoral degree
P9	Matron	50–55	30	18	Married	3	Bachelor’s degree
P10	Supervisor	50–55	26	15	Married	3	Bachelor’s degree
P11	Head Nurse	50–55	30	16	Married	2	Master’s degree

The analysis revealed that barriers and protective factors to decision fatigue among clinical nursing managers were shaped by a constellation of interrelated factors, encompassing managerial practices, communication dynamics, and individual cognitive and behavioral attributes. These influences coalesced into three overarching themes:

1. Organizational Decision‐Making and Leadership Strategies
2. Communication Management and Organizational Interactions
3. Individual and Organizational Patterns in Decision‐Making

Each category comprised two subcategories, which are detailed in Table 2.

**TABLE 2:** Main theme, categories, and subcategories influencing decision fatigue (barriers and protective factors) in nursing managers.

Main theme	Categories	Subcategories
Factors influencing decision fatigue (barriers and protective factors)	Organizational decision‐making and leadership strategies	‐ Decision‐making regulation and guidance ‐ Humanistic management and leadership
	Communication management and organizational interactions	‐ Activation of interaction and communication processes ‐ Utilization of support resources
	Individual and organizational patterns in decision‐making	‐ Independence and responsibility in decision‐making ‐ Organizational interest and commitment

## 3. Organizational Decision‐Making and Leadership Strategies

According to the participants’ statements, organizational decision‐making and leadership strategies were among the key factors contributing to decision fatigue in nursing managers during the COVID‐19 pandemic. According to the participants, effective decision‐making requires a combination of organizational structures and management characteristics. This category includes two subcategories of decision‐making regulation and guidance, and humanistic management and leadership.

### 3.1. Decision‐Making Regulation and Guidance

The participants believed that specialized training in decision‐making during a crisis increases the ability of managers to make optimal decisions.

Participant P3, a nursing manager in the 55–60 age range, stated that *during my management career, I saw people whose decisiveness and self-confidence increased after receiving training on decision-making and management solutions. Perhaps one reason for fatigue is the lack of required decision-making skills.* Another participant added:

*“When we received structured training on crisis decision-making, it felt like someone had finally given us a map. Without that, every decision felt heavier and more exhausting.” (P6)*

Participant P4, a head nurse in the 45–50 age range, stated that: *I set a goal for myself that I must reach, and this helped me resist pressure. I do not act hastily; I always determine priorities first.*

Another participant noted:

*“Having a roadmap meant I didn’t panic. Even when everything was chaotic, I knew what step came next, and that reduced my anxiety a lot.” (P9)*

Participant P4, a head nurse in the 45–50 age range, stated that: *When I wanted to make an important decision, I first implemented it as a pilot to identify advantages and obstacles, and then I implemented it. In this way, I could convince the senior manager that I am making a right decision.*

Another participant added:

*“Testing decisions on a small scale gave me confidence. It also prevented mistakes that could have caused more stress for the whole team.” (P2)*

A third participant emphasized:

*“Strategic thinking wasn’t optional during COVID. If you didn’t think three steps ahead, you were already behind, and that created enormous fatigue.” (P11)*

### 3.2. Humanistic Management and Leadership

The findings of this study reflect the experiences and perceptions of nursing managers regarding the factors influencing decision‐making during the COVID‐19 pandemic. Participants described that adopting a more humanistic and participatory leadership style helped them feel more supported and less overwhelmed in their decision‐making.

Participant P7, a head nurse in the 40–45 age range, stated that: *I once had a challenge with one of my employees, so the next day I went to the matron office and told her that I didn’t want this employee in my department anymore. She told me to talk to him and see why he reacted this way. My matron advised me to talk to him and understand the reason behind his reaction. She also told me to be a leader rather than just a manager*. *Her advice calmed me, and eventually that employee became one of the best in my department.*

*“When my supervisor listened to my concerns without judgment, it reduced half of my stress. Feeling understood made decision-making much easier for me.” (P10)*

*“Humanistic leadership meant my manager stood beside me, not above me. That sense of partnership prevented me from feeling overwhelmed.” (P1)*

The participants believed that managers who created effective motivational systems for their employees suffered less decision fatigue. They also emphasized that creating growth opportunities and paying attention to the psychological needs of employees play an important role in reducing decision fatigue in managers.

*“When I saw my staff motivated and emotionally supported, I felt less pressure. Their stability directly reduced my own decision fatigue.” (P8)*

*“Creating opportunities for staff development made them more independent. When they grew, I didn’t have to make every small decision myself.” (P8)*

## 4. Organizational Communication and Interaction Management

Participants described that communication and interaction within the organization shaped how they experienced decision‐making. They explained that clear communication channels, accessible supervisors, and supportive interactions helped them feel more coordinated and less isolated in their managerial roles. Participants also noted that transparent communication structures and opportunities for interaction made it easier for them to navigate complex decisions. This category includes two subcategories: activation of interaction and communication processes, and utilization of support resources.

### 4.1. Activation of Interaction and Communication Processes

The participants believed that factors such as improving management communication, having a hierarchical communication system and effective intraorganizational communication, encouraging interactions between employees, and establishing effective communication with stakeholders and external institutions can prevent managers from experiencing decision fatigue.

Participant P5, a supervisor in the 40–45 age range, stated that: With *all that hustle and bustle, the communication was good at the time of crisis and management was easier for me… For example, during that Delta crisis, we were really busy and patients were really sick, but the good communication of the nursing manager made management easier for me. That is how efficiently and easily it was done with empathy.*

*“When communication channels were clear, I didn’t waste energy chasing information. That alone reduced my fatigue significantly.” (P3)*

*“Encouraging staff to talk openly helped us solve problems faster. Silence and confusion were the real sources of exhaustion.” (P9)*

Participant P3, a nursing manager in the 55–60 age range, stated that: *During the Covid-19 pandemic, we provided a resting place for our nurses in a hotel…. We sought help from the nursing council. I suggested to consider a place under the responsibility of nursing council for patients who were in the recovery phase of their illness. They worked in convalescent homes for a long time and were helpful.*

*“Whenever we collaborated with external organizations, the workload felt lighter. Shared responsibility meant shared relief.” (P11)*

### 4.2. Utilization of Support Resources

According to the participants’ viewpoint, organizational support plays a major role in reducing the burden of decision‐making and increasing the managers’ efficiency. The participants believed that this support should cover three areas of strategic and managerial support, psychological support, and operational support, as well as resource provision.

Participant P6, a head nurse in the 35–40 age range, stated that: *To be honest, our senior supervisor was in line with me and did not stop me. I felt that she would reduce my fatigue and help me with planning*. *I felt supported.*

Psychological support and reduction of decision‐making pressure are factors that affect the promotion of managers’ mental and psychological health, which can include organizational stress reduction programs to improve decision‐making performance as an obstacle to decision fatigue. Operational support and resource provision refer to the creation of mechanisms that optimize administrative processes, reduce administrative barriers, and provide facilities and equipment to facilitate managers’ decisions.

*“Knowing that senior management backed my decisions gave me confidence. Without that support, every decision felt heavier.” (P4)*

Participant P2, a head nurse in the 35–40 age range, stated: *I changed a lot of places in order to move the ward’s equipment, because it was a large ward. For example, I expressed my opinions to the matron, saying that I should change the last two rooms that are not quite visible to store and put beds in the first two rooms to be used for patients, and she agreed.*

*“When resources were available, decisions were faster and clearer. Lack of equipment was one of the biggest sources of fatigue.” (P7)*

*“Operational support meant I didn’t have to fight for every small thing. That saved my energy for real decision-making.” (P5)*

## 5. Individual and Organizational Patterns in Decision‐Making

These patterns refer to a set of individual and organizational characteristics, behaviors, and structures that affect the way managers make decisions. The results showed that managers, who were assertive in their words, reduced dependency and increased managerial self‐confidence, experienced less decision‐making burden, and were able to make more efficient decisions during the pandemic. Also, managers who had a higher level of independence, delegated authority in a more appropriate way, and had organizational commitment were less likely to suffer from decision fatigue.

Participant P10, a supervisor in the 50–55 age range, stated: *For me, in order to avoid decision fatigue, it is very important to have a clear idea of how to make decisions in the moment and how much independence I have in the decisions I make.*

*“When I knew exactly what decisions I was authorized to make, I felt lighter. Unclear boundaries were the biggest source of fatigue for me.” (P4)*

*“Independence helped me trust my judgment. When I constantly had to seek approval, my energy drained quickly.” (P7)*

This category includes two subcategories of independence and responsibility in decision‐making, and organizational interest and commitment.

### 5.1. Independence and Responsibility in Decision‐Making

Independence and responsibility in decision‐making, as a determining factor, play a key role in reducing dependence on command structures and increasing managerial flexibility. Findings showed that managers who had a high level of intellectual independence were more capable of analyzing data, choosing efficient solutions, and implementing decisions in challenging situations. At the same time, responsibility, which includes accepting the consequences of decisions and committing to implementing them, increases self‐confidence and reduces anxiety caused by managerial pressures.

Participant P8, a supervisor in the 45–50 age range, stated: *In the meetings I was in, I spoke decisively, and since I have a long service history, I spoke in a way that led to what I wanted the meeting to go towards. For example, I ordered the bathroom and toilets in the children’s ward to be repaired and fixed, and resting room to be equipped.*

*“When I accepted responsibility for my decisions, I felt more confident and less anxious. Avoiding responsibility only increased my stress.” (P2)*

*“Independence didn’t mean working alone. It meant having the courage to make decisions and stand by them.” (P11)*

*“During COVID, hesitation was exhausting. Being decisive actually reduced my fatigue because it prevented constant back-and-forth.” (P6)*

### 5.2. Organizational Interest and Commitment

Participants described that having a sense of belonging and motivation toward the organization made them feel more confident and consistent when making and implementing decisions. They also noted that when they felt committed to the organization, interactions with colleagues became smoother and cooperation felt more natural in their daily managerial activities.

Participant P11, a head nurse in the 50–55 age range, stated: *I am very much interested in nursing profession. I am not in a great financial situation, but that is not my priority. Maybe it is my interest in work and management that motivates me, makes me not get tired and love my job.*

*“My commitment to the organization made me push through the hardest days. When you care about your workplace, fatigue feels different.” (P3)*

*“Even when resources were limited, my loyalty to the team motivated me to make the best decisions possible.” (P9)*

*“Managers who lack organizational attachment get tired faster. Interest in the job protects you from burnout.” (P1)*

## 6. Discussion

This study aimed to explore barriers and protective factors influencing decision fatigue among clinical nursing managers during the COVID‐19 pandemic. The findings of this study revealed that clinical nursing managers experienced decision fatigue as a result of a complex interplay between organizational, interpersonal, and individual factors during the COVID‐19 pandemic. Importantly, the results demonstrated that decision fatigue was shaped not only by barriers—such as organizational pressures, responsibility overload, and the need for rapid decision‐making under uncertainty—but also by protective factors, including supportive leadership, effective communication, and access to organizational resources. This dual perspective highlights both the sources of cognitive strain and the mechanisms that can mitigate it. Recognizing both dimensions underscores the importance of organizational strategies that reduce barriers while simultaneously strengthening protective mechanisms. Participants consistently reported that deficiencies in organizational decision‐making and leadership strategies heightened their experience of decision fatigue. The findings suggest that organizational structures and leadership approaches can either buffer or intensify decision fatigue depending on how they are enacted during crises. Participants highlighted the benefits of targeted decision‐making training and the implementation of pilot initiatives, which helped them maintain clarity during high‐pressure situations. These insights align with those of Dong et al., who observed increased decision fatigue among frontline nurses during health crises, underscoring the need for effective managerial interventions [20]. Managers who had predefined decision‐making roadmaps appeared to experience lower anxiety and greater efficiency during crisis conditions.

This is consistent with Sinaia et al., who emphasized the role of structured planning in reducing uncertainty and supporting managerial decision quality [28].

Another key contributor to decision fatigue identified in this study was the absence or presence of humanistic leadership. Participants reported that managers who adopted humanistic and participatory leadership styles experienced lower levels of decision fatigue and demonstrated more adaptive decision‐making. The findings suggest that trust‐based and participatory leadership styles may reduce decision fatigue by strengthening relational dynamics within teams. This aligns with Wang et al., who highlighted trust‐building and inclusive communication as essential to sound managerial decisions [29].

The present findings add nuance by showing that relational support from leaders helped managers feel more grounded and confident in their decisions. Taskiran et al. similarly found that participatory leadership styles improved job satisfaction and minimized decision fatigue among nursing staff, further validating our conclusions [30].

Communication management and organizational interactions also emerged as influential factors. Participants emphasized that effective communication with staff, peers, and superiors reduced psychological stress and improved the consistency of decision‐making. These findings echo those of Wang et al., who highlighted the stress‐reducing benefits of clear communication and collaborative consultation [29]. Interactive communication environments and collaborative structures appeared to support more efficient decision‐making.

Studies by Allan et al. further support this perspective, linking hierarchical and horizontal communication with reductions in administrative overload and improvements in managerial coordination [24]. Communication with external stakeholders also helped reduce operational challenges, as reflected in participants’ accounts.

Another study showed that clear communication and interorganizational interactions reduced work stress and improved managerial efficiency. These findings indicate that developing communication skills improves decision‐making and enhances coordination and cooperation in complex work environments. [25]. Our results reinforce the importance of removing communication barriers and promoting cross‐functional dialogue to reduce cognitive strain.

Organizational and managerial support structures, such as expert consultation and senior leadership backing, were also cited as buffers against decision fatigue. Participants reported increased self‐efficacy and confidence when such support was accessible. Pignatiello et al. confirmed that group decision‐making and clearly defined decision priorities help reduce managerial burden [31]. The findings suggest that strategic, psychological, and operational support structures may reduce decision complexity and enhance managerial confidence.

Lastly, individual and organizational patterns in decision‐making were highlighted. Managers with greater autonomy and delegated authority experienced less decision fatigue. These findings indicate that individual traits and organizational culture jointly shape decision quality and resilience during crises.

This supports findings by Goldsby et al., who reported enhanced decision flexibility and reduced dependence on hierarchical structures in environments with higher managerial autonomy. Organizational commitment was also identified as a protective factor, particularly in high‐stakes situations like the COVID‐19 pandemic [25].

The integration of individual capabilities with supportive organizational structures appears to enhance decision‐making processes and reduce decision fatigue.

As supported by Banisi’s commitment to the organization, it reduces job stress and conserves cognitive resources [32].

Collectively, these findings suggest that targeted leadership development, participatory communication structures, and supportive organizational frameworks are essential for managing decision fatigue among nursing managers, particularly during crises.

## 7. Conclusion

This study showed that decision fatigue among nursing managers during the COVID‐19 pandemic was shaped by both barriers and protective factors embedded within organizational and individual conditions, including leadership approaches, communication quality, institutional support, and levels of managerial autonomy. Structured decision‐making strategies—such as scenario planning, pilot testing, and clear communication pathways—helped reduce cognitive burden and improve decision quality.

Participatory and trust‐based leadership practices also emerged as important protective factors, supporting managerial resilience and enhancing performance under pressure. Based on these findings, healthcare organizations should adopt targeted measures to mitigate decision fatigue in high‐stress environments. Such measures include strengthening delegation systems, promoting collaborative decision‐making, providing psychological and organizational support, optimizing communication processes, and establishing flexible yet standardized guidelines for critical decisions.

Implementing these strategies can enhance leadership capacity, reduce managerial strain, and improve organizational responsiveness during future health crises. Further research is recommended to examine how leadership style, organizational resources, and psychological factors interact to influence decision fatigue among nursing professionals.

### 7.1. Strengths and Limitations

#### 7.1.1. Strengths

This study offers a comprehensive and multidimensional exploration of decision fatigue among nursing managers, identifying critical organizational and individual factors that influence this phenomenon.

• *Holistic Perspective*: The research examined decision fatigue through managerial, communicative, structural, and support‐oriented lenses, demonstrating how these interconnected domains contribute to or mitigate fatigue in managerial roles.
• *Alignment with Existing Literature*: By situating the findings within the context of prior studies, the research enhances its scientific credibility and broadens the interpretive framework for understanding decision fatigue.
• *Practical Implications*: The study presents actionable managerial and organizational strategies that can be applied in real‐world healthcare settings to improve decision‐making quality and reduce cognitive burden among nursing leaders.
• *Focus on Leadership and Communication*: The findings emphasize the significance of participatory leadership styles and effective organizational communication as key protective factors against decision‐related stress and diminished performance.

#### 7.1.2. Limitations

Despite its contributions, this study presents several limitations that should be acknowledged when interpreting the results:

• *Sampling Constraints*: The findings are based on a purposively selected group of nursing managers within specific institutional settings, which may limit the generalizability of results to broader populations.
• *Contextual Scope*: The research primarily investigated decision fatigue within the context of health crises, such as the COVID‐19 pandemic. As such, its applicability to routine or noncrisis conditions remains unexplored.
• *Lack of Longitudinal Insight*: The study did not assess the long‐term consequences of decision fatigue on organizational effectiveness or managerial mental health, which warrants further investigation in future research.

## Author Contributions

Sarah Sheikh and Maryam Esmaeili contributed to the conceptualization of the study. Mohammad Ali Cheraghi was responsible for the investigation. Fatemeh Hajibabaee developed and implemented the methodology. Maryam Esmaeili supervised the research process. Alireza Nikbakht Nasrabadi prepared the original draft and performed subsequent editing.

## Funding

This research received no specific grant from any funding agency in the public, commercial, or not‐for‐profit sectors.

## Disclosure

This study was conducted as part of a PhD dissertation in Nursing. All authors affirm full accountability for the integrity and accuracy of the research presented.

## Ethics Statement

All ethical principles were strictly adhered to throughout the research process. Prior to participation, all individuals received a detailed explanation of the study’s purpose and procedures and provided written informed consent. Participants were assured of the confidentiality of their responses, and no personally identifiable information was included in any reports or publications.

The study protocol received approval from the Research Council of Tehran University of Medical Sciences and was granted ethical clearance under the reference code IR.TUMS.FNM.REC.1401.129. Participants were informed of their right to withdraw from the study at any stage without any consequences.

## Consent

Please see the Ethics Statement.

## Conflicts of Interest

The authors declare no conflicts of interest.

## Data Availability

The data that support the findings of this study are available from the corresponding author upon reasonable request. Due to the nature of qualitative data and confidentiality agreements with participants, the full transcripts are not publicly available.
